# Natural killer cell exhaustion in ovarian cancer: from molecular suppression to therapeutic revival

**DOI:** 10.3389/fimmu.2025.1709075

**Published:** 2026-01-06

**Authors:** Jun Ning, Lan Yao

**Affiliations:** Department of general gynecology II, Gynecology and Obstetrics Center, the First Hospital of Jilin University, Changchun, China

**Keywords:** ovarian cancer, tumor microenvironment, natural killer cells, cytokines, immune checkpoints, metabolic reprogramming, NK cell-based immunotherapy

## Abstract

Ovarian cancer remains one of the most lethal gynecologic malignancies, largely due to its late-stage diagnosis and the establishment of an immunosuppressive tumor microenvironment (TME). Natural killer (NK) cells, key effectors of innate immunity, exhibit impaired cytotoxicity within this hostile niche. The dysfunction arises from multiple mechanisms, including suppression by immunosuppressive cytokines (TGF-β, MUC16), shedding of activating ligands (MICA/B, B7-H6, CD155), overexpression of inhibitory immune checkpoints (PD-1, TIGIT), and metabolic reprogramming shaped by glucose and lipid competition. Recent advances in NK cell–based immunotherapies—such as cytokine modulation, adoptive NK transfer, and checkpoint blockade—have demonstrated potential to reverse NK exhaustion and enhance antitumor efficacy. In this review, we systematically dissect the molecular and cellular pathways underlying NK cell suppression in ovarian cancer and evaluate emerging strategies to reinvigorate NK-mediated immunosurveillance.

## 1 Introduction

Ovarian cancer remains one of the deadliest malignancies affecting the female reproductive system, largely due to its insidious onset and frequent diagnosis at advanced stages, where symptoms are minimal or nonspecific. As a result, the five-year survival rate continues to hover below 30% (1). To overcome therapeutic resistance, maintenance regimens incorporating targeted agents—such as angiogenesis inhibitors and poly (ADP-ribose) polymerase (PARP) inhibitors—have demonstrated promising clinical efficacy (2). The tumor microenvironment (TME), particularly the immunologically active ascitic fluid in advanced ovarian cancer, has emerged as a critical factor in promoting immune evasion and facilitating tumor advancement. This microenvironment encompasses a diverse array of cancer cells, immune infiltrates, and soluble molecules, including cytokines and metabolites, that collectively suppress both innate and adaptive immune responses (3).

Among the innate immune components, natural killer (NK) cells play a pivotal role due to their ability to eliminate aberrant cells without prior sensitization and to coordinate immune modulation (4). However, the cytotoxic and regulatory functions of NK cells are frequently attenuated within the ovarian TME. Recent advancements in NK cell–based immunotherapies, such as adoptive NK cell infusion and CAR-modified NK platforms, offer potential to restore antitumor activity. This review focuses on elucidating the molecular and cellular mechanisms underlying NK cell dysfunction in ovarian cancer and highlights evolving therapeutic approaches aimed at restoring NK-mediated immunity.

## 2 Natural killer cell in ovarian cancer

Natural killer (NK) cells, pivotal constituents of the innate immune compartment, originate from the common lymphoid progenitors shared with B and T lymphocytes. As the first subclass identified within the innate lymphoid cell (ILC) family, NK cells exhibit robust cytolytic properties that mirror those of CD8^+^ T cells. Unlike their adaptive counterparts, NK cell activation is independent of antigen presentation; instead, it is orchestrated through the integration of activating and inhibitory signals transmitted via a repertoire of membrane-bound receptors (4). Under physiological conditions, the expression of major histocompatibility complex class I (MHC I) molecules on healthy cells engages inhibitory killer immunoglobulin-like receptors (KIRs), maintaining immune quiescence. In contrast, malignant transformation is often accompanied by MHC I downregulation, thereby attenuating inhibitory signaling and rendering tumor cells vulnerable to NK cell–mediated cytotoxicity. Simultaneously, cancer cells may express ligands for activating NK receptors, further tipping the balance toward immune activation and tumor clearance (5).

Phenotypically, NK cells are broadly categorized into two primary subsets based on CD56 and CD16 surface expression: CD56^brightCD16^-^ and CD56^dimCD16^+^ populations. The CD56^brightCD16^-^ subset, representing the dominant fraction within non-lymphoid tissues, is enriched in organs such as lymph nodes, liver, and kidneys. These immature NK cells exhibit minimal cytotoxicity but are prolific cytokine producers, releasing substantial quantities of immunoregulatory mediators, including interferon-γ (IFN-γ) and tumor necrosis factor-α (TNF-α) (6). In contrast, CD56^dimCD16^+^ NK cells—comprising nearly 20% of the NK cell compartment and 5–10% of peripheral blood mononuclear cells (PBMCs)—are specialized for direct cytolytic activity and dominate the circulatory pool. Upon engagement of CD16 with the Fc region of IgG antibodies, these cells initiate a signaling cascade via high-affinity Fcγ receptors, leading to phosphorylation of immunoreceptor tyrosine-based activation motifs (ITAMs) on CD3ζ chains and culminating in antibody-dependent cellular cytotoxicity (ADCC) (7). Once activated, NK cells establish an immunological synapse with their target, directing the focused release of cytotoxic vesicles containing perforin and granzymes. Perforin facilitates transmembrane pore formation, enabling granzyme entry into the cytoplasm, where they activate caspase pathways, thereby initiating apoptosis in the target cell (8).

## 3 NK cell function in the TME of ovarian cancer

### 3.1 Regulation of NK cell function via immunosuppressive cytokines

Cytokines, a class of small regulatory proteins secreted by both hematopoietic and non-hematopoietic cells in response to microenvironmental perturbations, play pivotal roles in modulating immune surveillance, cellular proliferation, and tissue integrity (7). In the context of ovarian cancer, the tumor microenvironment (TME) becomes enriched with immunosuppressive mediators, notably mucins and transforming growth factor-β (TGF-β), which significantly compromise the cytotoxic potential of natural killer (NK) cells. MUC16, commonly referred to as cancer antigen 125 (CA125), is an epithelial mucin that is extensively glycosylated and aberrantly expressed in ovarian malignancies. The membrane-anchored form (csMUC16) undergoes proteolytic cleavage, producing a soluble variant (sMUC16) that accumulates in ascitic fluid at concentrations ranging from 5 to 20 nmol/L and exerts direct inhibitory effects on NK cell function (9). Data from Belisle et al. (10) demonstrated that sMUC16 downregulates CD16 expression on NK cells by 40–70%, leading to marked attenuation of ADCC. Concurrently, csMUC16 interferes with the formation of immunological synapses between NK and tumor cells, thereby impeding effective cytotoxic engagement. Gubbels and colleagues (11) reported that NK cells more effectively eliminate ovarian cancer cells with low csMUC16 expression, which retain activating ligands for DNAM-1 and NKG2D receptors, highlighting MUC16’s critical role in immune escape. Furthermore, sustained exposure to MUC16 induces a phenotypic switch in NK cells from a cytolytic CD56^dimCD16^+^ subset toward a more immature CD56^brightCD16^-^ phenotype, further diminishing their tumoricidal capabilities.

Transforming growth factor-β (TGF-β), produced not only by malignant cells but also by immunosuppressive immune subsets such as regulatory T cells (Tregs), myeloid-derived suppressor cells (MDSCs), and tumor-associated macrophages (TAMs), plays a pivotal role in restraining NK cell activity in ovarian cancer (12). At the mechanistic level, TGF-β impairs NK cell metabolism by disrupting IL-2–dependent glycolytic flux, oxidative phosphorylation, and mTOR signaling driven by IL-15, collectively limiting both their expansion and effector functions (12). In the tumor ascites microenvironment, TGF-β reduces CD16 expression, thereby attenuating NK-mediated ADCC (13), and simultaneously inhibits the JAK-STAT and MAPK cascades essential for NK cell stimulation (14). Through transcriptomic profiling, Cortez et al. (15) demonstrated that TGF-β shifts NK cells toward a phenotype akin to innate lymphoid cells with reduced cytotoxic potential. Complementing these findings, Castriconi et al. (16) showed that exposure to TGF-β results in downregulation of activation receptors such as NKp30 and NKG2D. Collectively, the evidence implicates MUC16 and TGF-β as central regulators of NK cell dysfunction in ovarian carcinoma, emphasizing their value as immunotherapeutic targets to restore innate antitumor responses.

### 3.2 Interference with NK cell–activating receptor–ligand dynamics

Natural killer (NK) cell cytotoxicity is finely modulated by a balance between activating and inhibitory surface receptors. In the ovarian cancer tumor microenvironment (TME), malignant cells actively subvert this regulatory axis by expressing both membrane-bound and secreted forms of immunomodulatory ligands, thereby compromising NK cell surveillance (7). A well-characterized instance involves MHC class I–related molecules A and B (MICA/B), which serve as ligands for the activating receptor NKG2D, mediating cytolytic activity of NK cells (17). Within the TME, proteolytic enzymes such as matrix metalloproteinases (MMPs) and a disintegrin and metalloproteinases (ADAMs) cleave these ligands, producing soluble MICA/B (sMICA/B) isoforms. These soluble derivatives not only reduce the presence of membrane-tethered MICA/B but also function as decoy ligands, binding competitively to NKG2D, inducing receptor internalization, and impairing NK cell function (17, 18). To counter this evasion tactic, monoclonal antibodies targeting the α3 domain of MICA/B have been engineered to block proteolytic cleavage, maintaining ligand presentation on the cell surface. This strategy preserves NKG2D and CD16 expression and subsequently enhances NK cell–mediated cytotoxicity (19). Another evasion mechanism is mediated by B7-H6, a tumor-specific, stress-inducible ligand that engages NKp30, a potent activating receptor. In ovarian malignancies, B7-H6 is frequently found in a soluble form (sB7-H6), which persistently interacts with NKp30 and leads to receptor internalization and functional attenuation. This process not only diminishes NK cell–driven lysis but also suppresses the production of interferon-γ (20, 21). Importantly, B7-H6 expression is largely confined to ovarian, hematological, and breast malignancies, marking it as a promising cancer-specific biomarker for diagnostic and prognostic applications (22).

**Figure 1:**
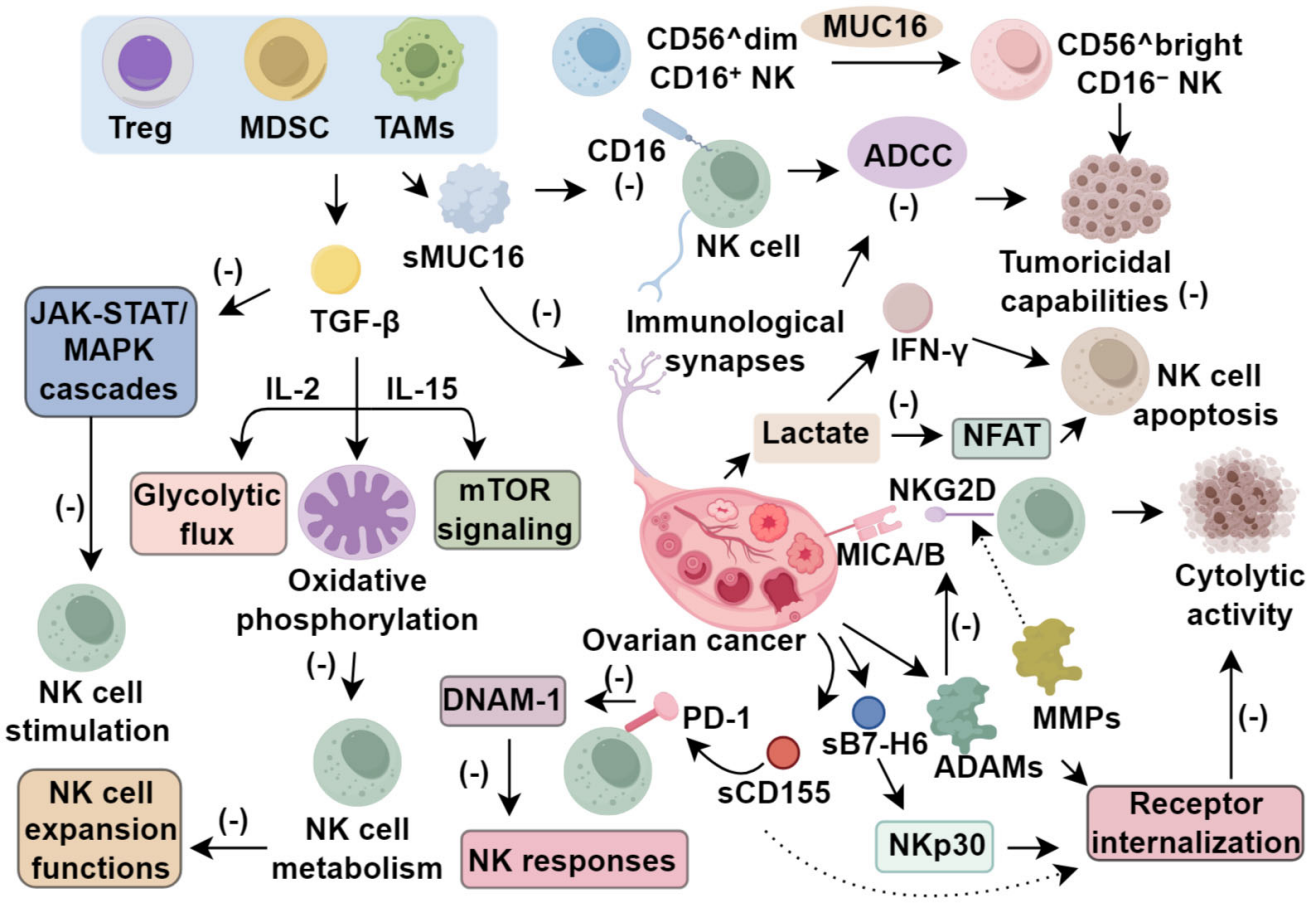
Function of NK cell in tumor microenvironment of ovarian cancer.

CD155, also referred to as the poliovirus receptor, functions as a shared ligand for both activating (DNAM-1) and inhibitory receptors (TIGIT, CD96), with its expression significantly upregulated across multiple tumor types, including ovarian cancer (23). Cellular responses to DNA damage and genotoxic stress stimulate the release of soluble CD155 (sCD155), whose elevated serum levels have been observed in patients with advanced gynecologic and breast malignancies, correlating with poorer prognosis (24, 25). Mechanistically, sCD155 impairs DNAM-1–mediated NK cell activation by promoting receptor internalization and degradation, ultimately suppressing NK cell cytotoxicity (26). In ovarian cancer, diminished DNAM-1 expression on NK cells recovered from ascitic fluid has been associated with adverse clinical trajectories, underscoring the critical immunological role of this pathway (27). The concerted shedding of activating ligands such as MICA/B, B7-H6, and CD155 exemplifies a convergent strategy through which ovarian tumors evade immune surveillance orchestrated by NK cells (**Figure 1**).

### 3.3 Exhaustion of NK cells in immune escape

To preserve immune tolerance and prevent overactivation, inhibitory checkpoint receptors such as PD-1 and TIGIT are essential regulators (28). However, tumors subvert these regulatory pathways to elude immune-mediated cytotoxicity. In the ovarian cancer microenvironment, NK cells upregulate multiple inhibitory receptors, undermining their anti-tumor effectiveness. While initially thought to be confined to T and B cells, PD-1 is now recognized as a negative regulator of NK cell activity—suppressing proliferation, dampening IFN-γ secretion, and impairing cytotoxicity. A dysfunctional, PD-1^+^ phenotype characterizes NK cells isolated from ascitic fluid, although this state can be partially reversed through checkpoint blockade (28, 29). When ovarian tumors lacking PD-L1 were implanted in murine models, enhanced NK infiltration and increased cytokine output were observed, alongside reductions in tumor burden and ascitic volume (30). Within the tumor niche, TIGIT expression is likewise elevated on NK cells, where it antagonizes DNAM-1 signaling due to its superior affinity for CD155 (31, 32). Rather than enabling co-stimulatory interactions, TIGIT binding suppresses NK responses. By disrupting DNAM-1 homodimer formation, TIGIT impedes downstream signaling required for CD155 engagement (33). Resistance to PD-1 monotherapy may result from this dual mechanism: TIGIT overexpression coupled with DNAM-1 suppression. Thus, rather than targeting PD-1 alone, co-inhibition of TIGIT and PD-1 may represent a more effective strategy to restore NK cell function and surmount tumor immune escape in ovarian cancer.

### 3.4 Metabolic reprogramming of NK cells in the ovarian cancer TME

Since the Warburg effect was first described, metabolic reprogramming has been recognized as a hallmark of cancer, enabling malignant cells to fulfill their high metabolic demands. Within the ovarian tumor microenvironment (TME), metabolites derived from tumor metabolism exert significant regulatory pressure on nearby immune cells, particularly NK cells. As tumors monopolize critical nutrients, NK cells are frequently subjected to metabolic insufficiency, leading to exhaustion and reduced cytotoxicity (34). Glucose, a fundamental energy substrate, is critical for both immune effector functions and cancer cell proliferation. While tumors predominantly utilize aerobic glycolysis, activated NK cells rely instead on oxidative phosphorylation (OXPHOS) to meet their energetic requirements (35). In ovarian malignancies, enhanced glycolytic activity increases both glucose consumption and lactate production, resulting in an acidic, nutrient-poor milieu. Lactate accumulation—facilitated by monocarboxylate transporter 4 (MCT4)—not only lowers extracellular pH but also enters NK cells, inducing intracellular acidification that suppresses NFAT signaling, diminishes IFN-γ secretion, and promotes apoptosis (36). *In vivo* studies by Brand et al. (37) confirmed lactate’s immunosuppressive effects; reducing its levels improved NK cell infiltration and suppressed tumor growth, effects absent in NK-deficient models. Additional insights from Assmann et al. (38) identified the citrate–malate shuttle and the sterol regulatory element-binding protein (SREBP) axis as central to NK cell metabolic adaptation. In the ovarian TME, the cholesterol metabolite 27-hydroxycholesterol—an endogenous SREBP inhibitor—is enriched, further impairing NK bioenergetic capacity and functional integrity.

While glucose metabolism remains integral to natural killer (NK) cell activity, lipid metabolism exerts equally pivotal influence over their functional competence. Within metastatic niches such as the omentum, tumor cells enhance fatty acid synthesis to sustain membrane biogenesis and pro-growth signaling pathways. This metabolic reprogramming, however, creates nutrient competition, wherein immune cells face restricted access to essential fatty acids due to altered transporter expression and local lipid scarcity. The resulting accumulation of lipid intermediates reconfigures the metabolic landscape to favor immune suppression (39). High expression of fatty acid synthase (FASN) correlates with unfavorable outcomes in ovarian cancer, and elevated circulating fatty acids are being investigated as diagnostic indicators (40). NK cells depend on fatty acid synthesis not only for membrane formation but also for clonal expansion during immune activation. Obesity exacerbates NK dysfunction; in both murine models and clinical observations, NK cells from obese individuals produce lower levels of IFN-γ, perforin, and granzyme B—hallmark molecules required for cytotoxic efficacy (41). This impairment is partly attributable to lipid accumulation regulated by peroxisome proliferator-activated receptors (PPARs), which suppress mTORC1 activity and consequently dampen glycolysis. PPAR agonists can simulate this suppression, thereby reducing both energy metabolism and cytolytic function in NK cells (42). Moreover, the formation of immunological synapses—a metabolically demanding process—is indispensable for NK-mediated killing. In the obese setting, NK cells rapidly lose membrane potential upon engaging tumor targets, reflecting a metabolic collapse that disrupts synapse stability and impairs effector responses (43). Thus, lipid-rich conditions in the ovarian tumor microenvironment, compounded by systemic obesity, critically undermine NK cell metabolic fitness and antitumor capacity.

## 4 Applications of NK cell immunotherapy in ovarian cancer

### 4.1 Cytokine-based modulation of NK cells

Multiple NK cell–centered immunotherapeutic strategies—ranging from cytokine stimulation and adoptive transfer to immune checkpoint inhibition and monoclonal antibody–based regimens—have been explored in ovarian cancer (44). Among them, preconditioning NK cells with interleukin-2 (IL-2) and interleukin-15 (IL-15) has shown promise in enhancing both their expansion and cytolytic performance against tumor cells (45). Rather than systemic delivery, intraperitoneal IL-2 administration has demonstrated favorable tolerance and pronounced NK cell proliferation in clinical trials (46). IL-15 elicits similar effects, augmenting not only NK cell count but also their effector potential. For example, Felices et al. (47) demonstrated that either monomeric IL-15 or its superagonist ALT-803 restores NK cell cytotoxicity in ascites-derived populations by upregulating IFN-γ and TNF-α secretion. In the tumor environment, chemokines act as key regulators of immune cell dynamics. Elevated levels of CXCL9, CXCL10, and CXCL11 in ascitic fluid facilitate NK cell trafficking to tumor sites and are associated with impaired tumor progression (48). These findings suggest that intraperitoneally administered cytokine regimens can reinvigorate dysfunctional NK cells within the ovarian cancer milieu. Nevertheless, early-phase clinical investigations targeting cytokine pathways have yielded variable efficacy—likely a consequence of the substantial tumor burden present at therapy initiation, which may blunt the response to immune modulation.

### 4.2 Adoptive transfer strategies for NK cell–mediated immunotherapy

Adoptive NK cell therapy entails harvesting peripheral blood NK cells, expanding and functionally enhancing them ex vivo, and subsequently reinfusing them either autologously or allogeneically to potentiate tumor-targeting immunity. To boost therapeutic specificity and efficacy, NK cells can be engineered to express chemokine receptors, co-stimulatory domains, or chimeric antigen receptors (CARs), thereby enabling antigen-directed cytotoxic responses (49). While CAR-T therapy has demonstrated substantial success against refractory hematological malignancies, its performance in solid tumors remains limited, largely due to insufficient tumor infiltration and risks such as cytokine release syndrome. In contrast, CAR-engineered NK cells offer a more favorable safety and efficacy profile. Progress in CAR-NK platforms has led to several clinical investigations in solid tumors, with promising outcomes. One notable example is FT536, a CAR-NK product derived from allogeneic induced pluripotent stem cells (iPSCs), which targets the MICA/B α3 domain and has received FDA investigational new drug approval for solid tumor treatment, including ovarian cancer (50). CAR-NK cells co-expressing the activating receptor NKG2D and the co-stimulatory molecule 2B4 have shown improved cytotoxic potential and enhanced clearance of mesothelin (MSLN)-positive ovarian tumor xenografts (51). Moreover, the study by Klapdor et al. (52) demonstrated synergistic cytotoxicity when combining anti-CD133 CAR-NK cells with cisplatin, suggesting that combinatorial strategies may further augment therapeutic efficacy. Despite these advances, adoptive CAR-NK therapy still faces logistical and technical hurdles—including suboptimal ex vivo proliferation, activation, transduction, cryopreservation, and transport. Overcoming these bottlenecks is critical to fully unlocking the clinical utility of CAR-NK–based approaches in solid tumors.

### 4.3 Modulation of NK cell activity

The cytotoxic behavior of natural killer (NK) cells is orchestrated through complex ligand–receptor networks. Tumor cells frequently exploit this system by expressing higher levels of inhibitory ligands, thereby attenuating NK activation. Although immune checkpoint inhibitors were originally developed to reinvigorate T cell responses, they have now been shown to potentiate NK cell activity as well. Agents targeting checkpoints like PD-1/PD-L1 and CTLA-4 can disrupt suppressive interactions on NK cells, thereby restoring their effector function and limiting tumor growth and dissemination (53). In preclinical models of peritoneal ovarian cancer, ablation of the PD-L1 gene led to marked reductions in tumor mass and ascites. This genetic deletion was accompanied by an expansion of circulating NK and T cell populations and increased secretion of proinflammatory mediators, including IFN-γ, TNF-α, IL-12, IL-15, CX3CR1, and CXCR1 (30). Interestingly, IFN-γ not only contributes to antitumor responses but also upregulates PD-L1 on tumor-infiltrating NK cells, forming a feedback loop. However, this can be counteracted by PD-L1–targeting antibodies, which further amplify NK cytotoxicity by enhancing IFN-γ and TNF-α release. Combining PD-L1 blockade with cytokines that promote NK activation has produced superior antitumor effects compared to either approach alone. These findings underscore the therapeutic potential of integrating checkpoint inhibition with NK-directed stimulation to enhance clinical outcomes in solid malignancies.

## 5 Conclusion

NK cells are essential players in ovarian cancer immunosurveillance but become progressively dysfunctional within the immunosuppressive tumor microenvironment. Factors such as TGF-β signaling, immune checkpoint upregulation, ligand shedding, and metabolic competition act in concert to dampen NK cell cytotoxicity and cytokine production. These alterations not only facilitate tumor immune evasion but also contribute to disease progression and therapeutic resistance. Understanding the interplay between ovarian cancer cells and NK cell-inhibitory pathways provides a mechanistic framework for identifying novel immunotherapeutic targets.

Therapeutic innovations aiming to restore NK cell function—ranging from cytokine-based strategies (IL-15 superagonists), adoptive NK cell therapies, CAR-NK constructs, to combined immune checkpoint blockade—are rapidly evolving. Preclinical and early clinical studies show that integrating these modalities can synergistically enhance NK effector responses and improve treatment outcomes in ovarian cancer. However, translating these findings into durable clinical benefit requires overcoming challenges related to NK cell persistence, trafficking, and tumor infiltration. Future research should focus on optimizing NK cell engineering, mitigating TME-induced suppression, and identifying biomarkers for patient stratification to guide personalized NK-based therapies.
